# An electronic device based on gold nanoparticles and tetraruthenated porphyrin as an electrochemical sensor for catechol

**DOI:** 10.1098/rsos.170675

**Published:** 2017-12-20

**Authors:** Luana M. Sousa, Luana M. Vilarinho, Gabriel H. Ribeiro, André L. Bogado, Luís R. Dinelli

**Affiliations:** 1 Faculdade de Ciências Integradas do Pontal, Universidade Federal de Uberlândia, Rua vinte, 1600, CEP 38304-402, Ituiutaba, Minas Gerais, Brazil; 2 Departamento de Química, Universidade Federal de São Carlos, CP 676, CEP 13565-905, São Carlos, Sao Paulo, Brazil

**Keywords:** dihydroxybenzene, electrode modified, electropolymerization, cobalt porphyrin

## Abstract

The aim of this study was to obtain an electrochemical device between the electrostatic interaction of the electropolymerized porphyrin {CoTPyP[RuCl_3_(dppb)]_4_}, where TPyP = 5,10,15, 20-tetrapyridilphorphyrin and dppb = 1,4-bis(diphenylphosphino)butane, and gold nanoparticles (AuNPs^n−^), to be used as a voltammetric sensor to determine catechol (CC). The modified electrode, labelled as [(CoTPRu_4_)_n_^8+^-BE]/AuNPs^n−^ {where BE = bare electrode = glassy carbon electrode (GCE) or indium tin oxide (ITO)}, was made layer-by-layer. Initially, a cationic polymeric film was generated by electropolymerization of the {CoTPyP[RuCl_3_(dppb)]_4_} onto the surface of the bare electrode to produce an intermediary electrode [(CoTPRu_4_)_n_^8+^-BE]. Making the final electronic device also involves coating the electrode [(CoTPRu_4_)_n_^8+^-BE] using a colloidal suspension of AuNPs^n−^ by electrostatic interaction between the species. Therefore, a bilayer labelled as [(CoTPRu_4_)_n_^8+^-BE]/AuNPs^n−^ was produced and used as an electrochemical sensor for CC determination. The electrochemical behaviour of CC was investigated using cyclic voltammetry at [(CoTPRu_4_)_n_^8+^-GCE]/AuNPs^n−^ electrode. Compared to the GCE, the [(CoTPRu_4_)_n_^8+^-GCE]/AuNPs^n−^ showed higher electrocatalytic activity towards the oxidation of CC. Under the optimized conditions, the calibration curves for CC were 21–1357 µmol l^−1^ with a high sensitivity of 108 µA µmol l^−1^ cm^−2^. The detection limit was 1.4 µmol l^−1^.

## 1. Introduction

Catechol (CC) is a phenolic compound commonly used in the cosmetic industry, for tanning, flavouring agents, pesticides, medicines, antioxidant, dye, photography, etc [1]. The lethal human dose of CC is 50–500 g kg^−1^ or one teaspoon given once to a person weighing 70 kg and death results from pulmonary failure [2]. Therefore, it is important to establish a sensitive, fast and convenient method for the detection and quantification of CC. Various techniques including liquid chromatography [3], capillary electrochromatography [4], electrochemiluminescence [5] and spectrophotometry [6] have been used to detect polyphenols. As CC presents excellent electrochemical processes, there are various electrochemical methods for its determination which can be found in the literature [7–15]. Compared with other methods, electrochemical methods have advantages such as being simple, sensitive and selective for samples with small concentrations.

Many materials involving metals, various nanotubes and nanoparticles, graphene and fullerene and porphyrin and their derivatives have been extensively used to construct electrochemical sensors [16–30]. Considering this, porphyrin, metalloporphyrin and their derivatives, have received much attention as they show excellent electrocatalytic properties [31]. Moreover, porphyrins and their metal derivatives are potential candidates for the construction of electrochemical sensors and catalytic applications as they present an extensively conjugated two-dimensional π-system and special electrochemical properties [32,33].

In addition, gold nanoparticles (AuNPs^n−^) have aroused considerable interest in applications as electrochemical sensors because they are easily synthesized and manipulated [34–38]. AuNPs^n−^ has a unique optical and electrochemical property that significantly depends on its morphological and physiological characteristics, i.e. on its size, shape and aggregation state and can be easily controlled by the synthesis methodology [39,40]. In addition, because nanoparticles have a large volume/surface ratio, when placed on the surface of the electrode they can increase their effective area and, owing to the spherical morphology, the interstitial space allows for an efficient mass transfer. Regarding the metallic nature of the nanoparticles, the conductivity of the electrode can be increased, improving its sensitivity and selectivity [41,40]. In recent years, interest in the study of aggregates of AuNPs^n−^ with ruthenium complex, labelled as [Ru]^+^/AuNPs^n−^, which takes place through a self-assembly process owing to the electrostatic interaction between species, has increased. These aggregates have been used in electrochemical devices [42,43] and homogeneous catalysis [44]. As a result, improvements in electrochemical responses, such as sensibility, stability, selectivity and catalytic activity, have been observed in the presence of AuNPs^n−^.

Many efforts have been made to develop modified electrodes using the electropolymerization of tetraruthenated porphyrin and use them as electrochemical sensors for phenolic compounds [45–47]. The biggest advantage of using electropolymerization is that it controls the number of layers deposited on the surface of a bare electrode (BE), such as glassy carbon electrode (GCE) or indium tin oxide (ITO). This process can be controlled by voltammetric cycles, and consequently the polymeric film thickness formed on the electrode. Recently, the simultaneous determination of CC and hydroquinone (HQ) was reported using a modified GCE with the {VOTPyP[RuCl_3_(dppb)]_4_} porphyrin [46]. In this study, it was demonstrated that the modified electrode (VOTPRu-GCE) improves the electrochemical response of CC and HQ when compared with the bare GCE. The electrochemical behaviours of the two isomers were reversible at the VOTPRu-GCE with two pairs of well-defined voltammetric peaks for the electrochemical processes of the analytes. Furthermore, the redox peak currents and sensitivity in the determination of CC and HQ were also greatly enhanced compared to the BE, 12.73 and 15.91 µA µmol L^−1^ cm^−2^, respectively, at VOTPRu-CGE. These results suggest that the electropolymerized film on the GCE surface can accelerate the electron transfer between analytes and electrode, which is probably caused by a synergistic effect of the vanadium ion in the centre of a porphyrin ring with the complexes containing ruthenium at the peripheral position.

The interaction and synergistic effect with layer–layer assembly of [(CoTPRu_4_)_n_^8+^-BE]/AuNPs^n−^ {where BE = bare electrode = GCE or ITO} and AuNPs^n−^ = gold nanoparticles was investigated. These electronic devices were characterized by UV/Vis measurements and atomic force microscopy (AFM) images and they were used as an electrochemical sensor for CC. The [(CoTPRu_4_)_n_^8+^-GCE]/AuNPs^n−^ showed an outstanding performance in the determination of CC in 0.1 mol l^−1^ acetate buffer at pH 4.76. The viability of the sensor was evaluated, such as the linear range, detection limit, sensitivity, stability and reproducibility.

## 2. Material and methods

### 2.1. Materials

CC was purchased from Vetec and all the chemicals used were of reagent grade or comparable purity. Methanol, dichloromethane and ether were distilled prior to use. All the aqueous solutions were prepared with ultra-pure water (ASTM type I, 18 MΩ cm of resistivity). Ruthenium(III) chloride hydrate, 1,4-bis(diphenylphosphino)butane (dppb), chloroauric acidH [AuCl_4_], tetrabutylammonium hexafluorophosphate (TBAH) and cobalt (II) acetate salt were purchased from Aldrich and they were used as received. Acetate buffer solutions (HAc-NaAc, 0.1 mol l^−1^) with different pH values were prepared by mixing 0.1 mol l^−1^ of acetic acid (HAc) and 0.1 mol l^−1^ of sodium acetate (NaAc) and the pH was adjusted by NaOH or HCl. Purified argon atmosphere was used in all procedures described to remove the dissolved oxygen.

### 2.2. Apparatus

Voltammetric experiments were performed on a µAUTOLAB type III potentiostat/galvanostat with a conventional three-electrode system. A bare or modified GCE (geometric diameter = 0.2 cm) or ITO electrode was used as a working electrode. A KCl saturated Ag/AgCl (AgCl_(sat)_) electrode was used as a reference electrode and a platinum wire as a counter electrode. All the experiments were carried out at room temperature. The cyclic voltammetry (CV) was performed from −0.4 to 1.0 V versus Ag/AgCl_(s)_ with a scan rate of 100 mV s^−1^, sample interval, 0.0001 V and quiet time of 2 s. Under the conditions used, E_0_ for the one-electron oxidation of [Fe(*η*^5^-C_5_H_5_)_2_], added to the test solutions as an internal standard, is +0.43 V.

UV/Vis spectra were recorded on a Shimadzu spectrophotometer, model UV-1800, coupled with a thermoelectrically temperature controlled cell TCC-100 (at 25 ± 0.1°C), using a quartz cell (1 cm), between 200 and 800 nm. Elemental analyses were performed with a Thermo Scientific CHNS-O FLASH 2000 micro analyzer. AFM measurements were performed using a Nanoscope V Veeco/Bruker with a scan assist.

### 2.3. Preparation of gold nanoparticles

AuNPs^n−^ with an average diameter of 5 nm were prepared by reduction of H[AuCl_4_] in a biphasic mixture of water/toluene (1 : 1) with sodium citrate tribasic dihydrate and sodium tetrahydridoborate. Hexadecyltrimethylammonium bromide (HDTBr) was used as a transfer reagent phase by modifying a procedure described in the literature [48]. In brief, 1.0 ml of 1% H[AuCl_4_] was added to 90 ml of H_2_O at room temperature (20–23°C). After 1 min of stirring, 2.0 ml of sodium citrate tribasic dihydrate (38.8 mmol l^−1^) was added. The solution was stirred for another 1 min, and then 1.0 ml of fresh 0.075% NaBH_4_ was added to the solution of sodium citrate tribasic dihydrate (38.8 mmol l^−1^). After this, 20 ml of HDTBr in toluene solution (0.13 mol l^−1^) was mixed with aqueous AuNPs^n−^. The resulting solution was kept under stirring for 20 min to move the AuNPs^n−^ swiftly from an aqueous phase to an organic phase. Afterwards, the colloidal solution was allowed to stand for phase separation, and then the mixture was separated using an analytical funnel and the organic phase was stored in a dark bottle at 4°C.

### 2.4. Synthetic procedures

The 5,10,15,20-tetra(4-pyridyl)porphyrin (H_2_TPyP) and 5,10,15,20-tetra(4-pyridyl)porphyrincobalt(II) [CoTPyP] were synthesized according to procedures reported in the literature [49]. The synthesis of the {CoTPyP[RuCl_3_(dppb)]_4_} complex was performed by modifying a procedure described in the literature [47]. Six milligrams (8.88 µmol) of [CoTPyP] [50,51] and 23.1 mg (3.55 µmol) of *mer*-[RuCl_3_(dppb)H_2_O] [52] were solubilized in a mixture of 27 ml of dichloromethane and 3 ml of methanol. The solution was stirred for 20 h and the resulting mixture was concentrated to about 3 ml. Then, Et_2_O (20 ml) was added to precipitate a brown-red solid. The complex was collected, washed with Et_2_O (three times, 10 ml) and dried under vacuum.

### 2.5. Preparation of the modified electrode

The modified electrode was obtained by modifying a procedure described in the literature [46]. Initially, the GCE surface was polished in alumina slurry (3 µm) and washed with ultra-pure water in an ultrasonic bath. The following procedure was performed to produce the first bilayer film: the electropolymerization of tetraruthenated porphyrin on the GCE surface was carried out in dichloromethane solution (0.1 mol l^−1^ TBAH used as a supporting electrolyte) of 0.1 mmol l^−1^ {CoTPyP[RuCl_3_(dppb)]_4_} by one voltammetric cycle in the range of potential between −0.4 and + 1.0 V at a scan rate of 100 mV s^−1^. The modified GCE [(CoTPRu_4_)_n_^8+^-GCE] was washed with acetone and ultra-pure water and was then added to a solution for 5 min containing 10 ml of gold nanoparticles (AuNPs^n−^, 0.05 mol l^−1^ of Au). The new modified electrode, labelled as [(CoTPRu_4_)_n_^8+^-GCE]/AuNPs^n−^, was washed with water to remove the excess of non-immobilized AuNPs^n−^. This procedure was repeated threefold before applying the [(CoTPRu_4_)_n_^8+^-GCE]/AuNPs^n−^ as a work electrode. The [(CoTPRu_4_)_n_^8+^-GCE]/AuNPs^n−^ was conditioned with 50 voltammetric cycles in NaAc solution (0.1 mol l^−1^) to stabilize the current and potential. The same procedure was used to build the electrode on the ITO surface. The ITO electrode was not used for electrochemical measurements of CC because it has low electrical conductivity.

## 3. Results and discussion

### 3.1. Preparation and characterization of the modified electrode

**Scheme 1.**
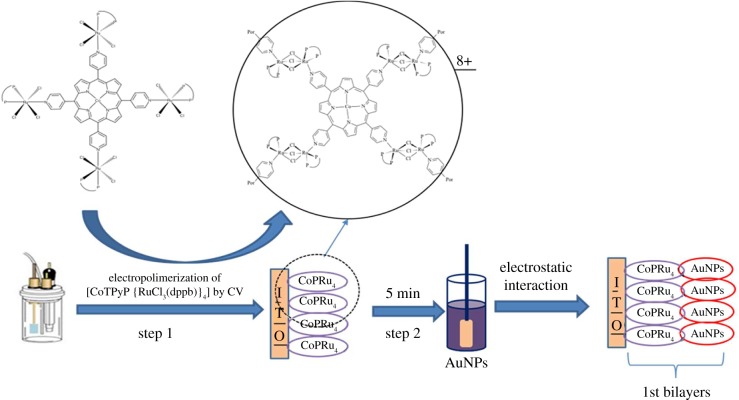
Formation of film on the surface of the ITO electrode in two steps.

The preparation of the modified electrode, labelled as [(CoTPRu_4_)_n_^8+^-BE]/AuNPs^n−^ {where BE = bare electrode = GCE or ITO}, was done layer-by-layer in two basic steps (see scheme 1). The first step requires the electropolymerization, by CV, of the porphyrin {CoTPyP[RuCl_3_(dppb)]_4_} on the surface of the electrode, making a voltammetric cycle in the potential range between −0.4 and + 1.0 V at a scan rate of 100 mV s^−1^. The second step involves a coating of the electropolymerized porphyrin with a colloidal solution of AuNPs^n−^.

**Figure 1.**
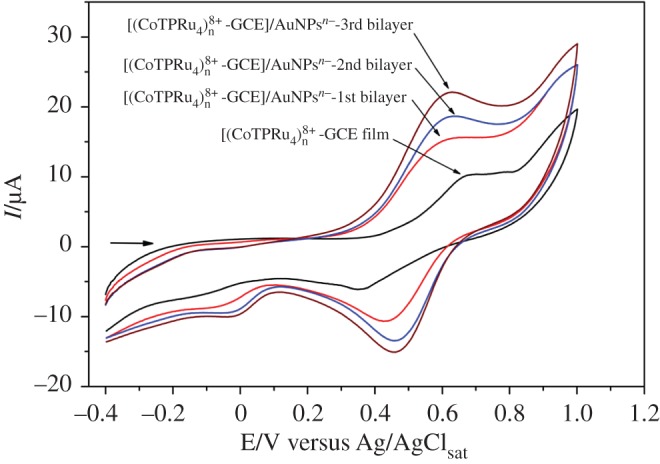
Electropolymerization process of 1 mmol l^−1^ {CoTPyP[RuCl_3_(dppb)]_4_} in dichloromethane (0.1 mol l^−1^ TBAH) on ITO electrode for each bilayer [(CoTPRu_4_)_n_^8+^-ITO]/AuNPs^n−^ by voltammetric cycles in the range of −0.4 to 1.0 V at 100 mV s^−1^.

The electropolymerized porphyrin produces a cationic species of mixed-valence {CoTPyP[Ru(dppb)_4_(Cl_3_)_2_}_2n_^8*n*+^, which adsorbs on the electrode surface (figure 1). The mechanism of the electropolymerization, including all characterizations of the film, was previously published by our research group [52] and involves the reduction of ‘RuCl_3_(dppb)' moiety (Ru^III^→Ru^II^) at 0 V. The quasi-reversible redox process of Ru^II^Ru^III^→Ru^III^Ru^III^ couple is observed at E_1/2_ = 0.55 V, assigned to the formation of the films of the mixed-valence complex {CoTPyP[Ru(dppb)_4_(Cl_3_)_2_}_2n_^8*n*+^. The aggregation of AuNPs^n−^ on the cationic polymeric film occurs by physical adsorption using electrostatic interactions [42,44].

**Figure 2.**
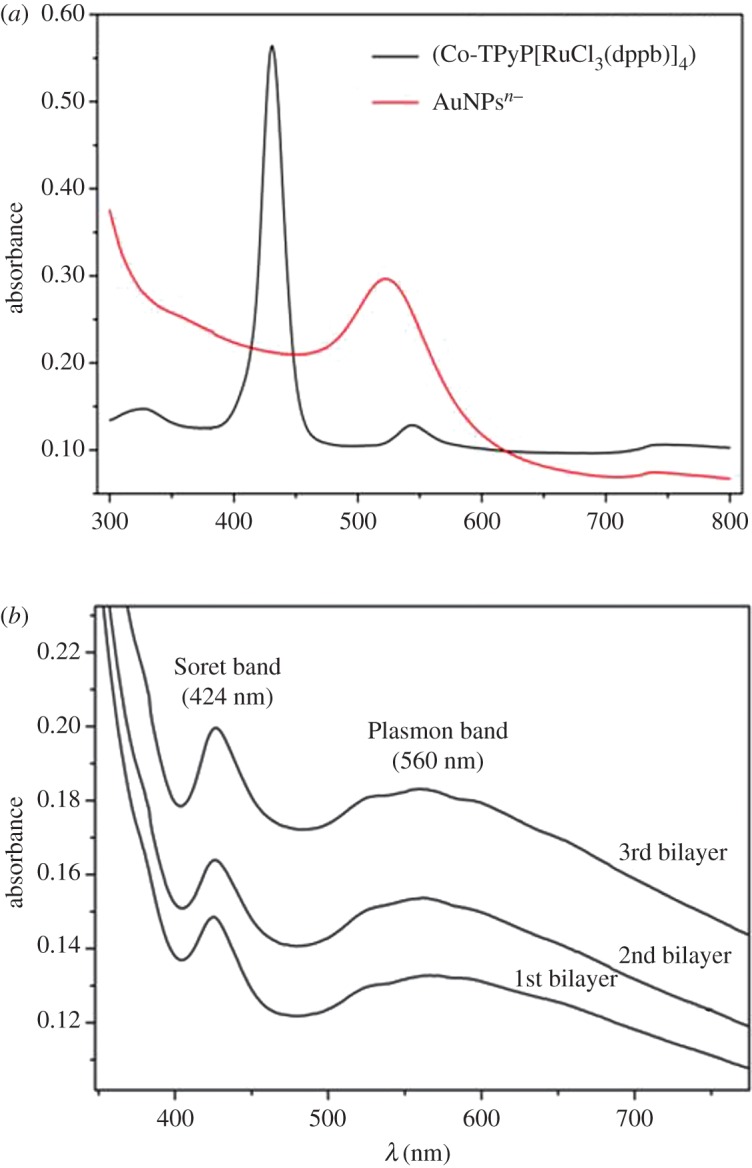
(*a*) UV/Vis spectrum of [CoTPyP{RuCl_3_(dppb)}_4_] in CH_2_Cl_2_ solution and AuNPs^n−^ in toluene solution; (*b*) monitoring of film formation of [(CoTPRu_4_)_n_^8+^-ITO]/AuNPs^n−^ by electronic spectrum onto ITO surface.

The two basic steps described above were carried out threefold, which produced a total of three bilayers, that presented the optimal condition for CC sensing. In figure 1, it can be observed that the peak currents increase with the number of cyclic voltammograms, owing to the increase in the immobilized species on the electrode surface. Figure 2*a* shows the UV/Vis spectra of the AuNPs^n−^ in the toluene solution and [CoTPyP{RuCl_3_(dppb)}_4_] in the CH_2_Cl_2_ solution. The Soret band of porphyrin and Plasmon band of AuNPs^n−^ with a wavelength at 425 and 525 nm, respectively, can be observed. In figure 2*b*, the UV/Vis spectra after the formation of each bilayer reveal the presence of Soret and Q bands, related to the electropolymerized porphyrin and a Plasmon band centralized at 530 nm, related to the AuNPs^n−^. These results show that the two species are immobilized onto the surface of the ITO electrode, producing the desired modified electrode labelled as [(CoTPRu_4_)_n_^8+^-ITO]/AuNPs^n−^.

**Figure 3.**
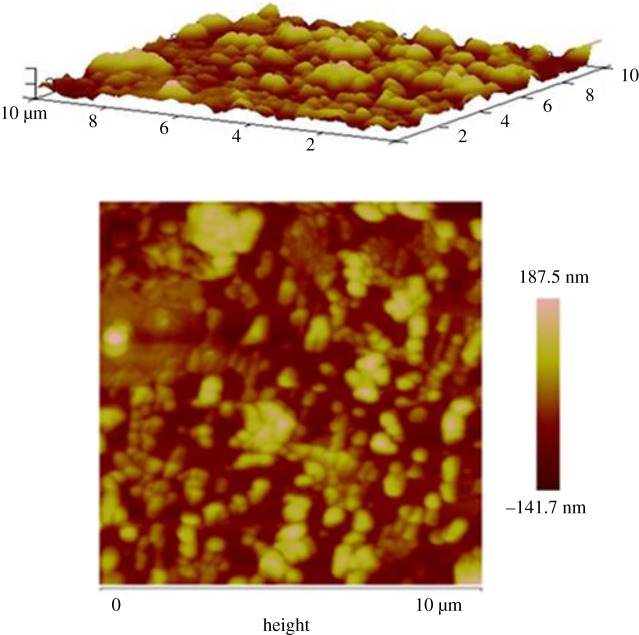
AFM image of a [(CoTPRu_4_)_n_^8+^-ITO]/AuNPs^n−^ film deposited onto ITO electrode surface.

AFM also characterized the modified electrode [(CoTPRu_4_)_n_^8+^-ITO]/AuNPs^n−^ (figure 3). The AFM image shows an average roughness of 54.5 nm in an area of 107 µm^2^, when three bilayers were bonded onto the ITO surface. This value is higher than the electropolymerized porphyrin [Mn(H_2_O)_2_TPyP{RuCl_3_(dppb)}_4_](PF_6_), which is a similar system without AuNPs^n−^, with a roughness value of 4.5 nm, even after five cyclic voltammograms [45]. This result shows that the immobilization of the AuNPs^n−^ on the surface of the electrode causes an increase in the roughness of the film. Undoubtedly, the increasing electroactive area is directly associated with the presence of AuNPs^n−^.

### 3.2. Electrochemical behaviour of catechol

**Figure 4.**
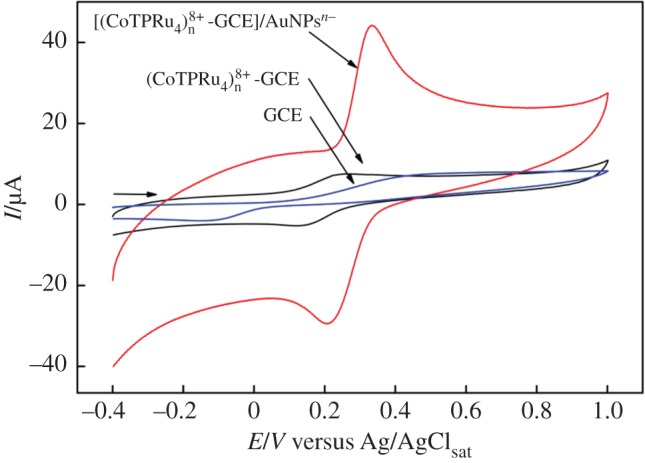
Cyclic voltammograms of 0.30 mmol l^–1^ CC in 0.1 mol l^–1^ acetate buffer solution (pH 4.76) at the [(CoTPRu_4_)_n_^8+^-GCE]/AuNPs^n−^; [(CoTPRu_4_)_n_^8+^-GCE] and GCE.

Efforts were made in order to evaluate the [(CoTPRu_4_)_n_^8+^-BE]/AuNP_s_^n−^ {where BE = bare electrode = GCE or ITO} as a voltammetric sensor for CC. The best results were obtained when GCE was used as a holder electrode and the performance of [(CoTPRu_4_)_n_^8+^-GCE]/AuNPs^n−^ was compared with the [(CoTPRu_4_)_n_^8+^-GCE] and nude GCE. Figure 4 shows the cyclic voltammograms of CC (0.30 mmol l^−1^), using the nude GCE as a work electrode, exhibiting the anodic peak potential (*E*_pa_) at 516 mV and cathodic peak potential (*E*_pc_) at −126 mV. The potential peak difference (*ΔE*_p_) was 642 mV, which means that the oxidation of the CC is an irreversible process in the uncovered GCE. However, the work electrode was altered to the [(CoTPRu_4_)_n_^8+^-GCE], the electrochemical behaviour of the CC was changed from irreversible to quasi-reversible, with the *E*_pa_ at 251 mV and *E*_pc_ at 126 mV, and a significant decrease in the *ΔE*_p_ = 125 mV. Finally, in the third circumstance, when the [(CoTPRu_4_)_n_^8+^-GCE]/AuNPs^n−^ was used as a sensor for CC detection, well-resolved redox processes were also observed, *E*_pa_ = 334 mV; *E*_pc_ = 209 mV; and *ΔE_p_* = 125 mV, related to the exposed GCE. The main advantage of AuNPs^n−^ on the electrode was a significant increase in the peak currents (*I*_p_), which was approximately seven times higher than the *I*_p_ obtained with the [(CoTPRu_4_)_n_^4+^-GCE]. These results show that the AuNPs^n−^ increases the sensitivity of the electrode.

### 3.3. Influence of pH and scan rate

**Figure 5.**
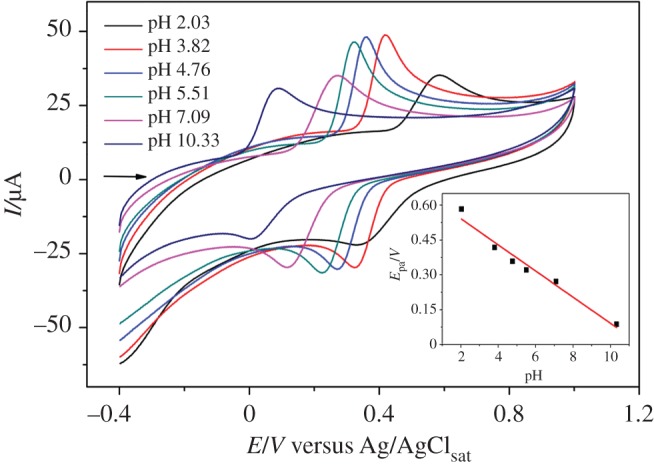
Cyclic voltammograms of 0.33 mmol l^−1^ CC at [(CoTPRu_4_)_n_^8+^-GCE]/AuNPs^n−^ in 0.1 mol l^−1^ acetate solution with different pH. Inset: effect of pH on the anodic peak potential.

The effect of the pH solution and scan rate on the electrochemical behaviour of CC with [(CoTPRu_4_)_n_^8+^-GCE]/AuNPs^n−^ as a work electrode was investigated by CV. Figure 5 shows the cyclic voltammograms of CC (0.33 mmol l^−1^) performed at different pH solutions in the range of 2.0–10.3. The acetate buffer solution (pH 4.76) provides the optimum anodic peak current (*I*_pa_), *E*_1/2_ and *ΔE*_p_ among the investigated conditions. Consequently, the acetate buffer solution was chosen as the supporting electrolyte in the protocol of the quantitative analyses.

The influence of the pH solution on the peak potentials of CC was also studied. It was observed that the *E*_pa_ of CC decreases with the increase of the pH. A linear relationship can be noted between the *E*_pa_ and pH, described as follows (equation (3.1)):

3.1 $$E_{\mathrm{pa}}(\text{mV})=-52.5\text{ pH}+0.64\;(R^{2}=0.885).$$

**Figure 6.**
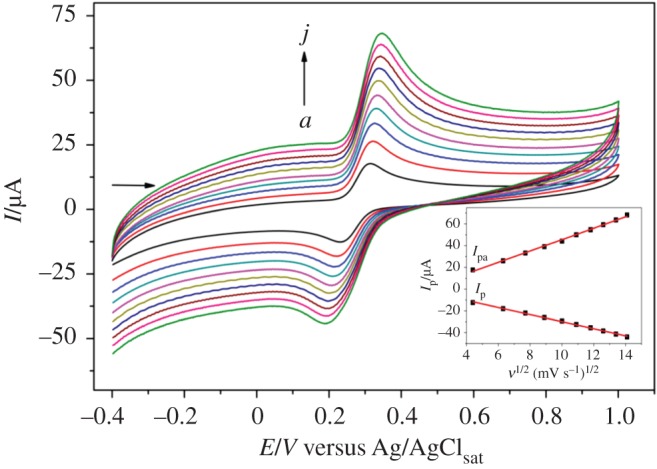
Cyclic voltammograms of 0.30 mmol l^−1^ CC in 0.1 mol l^−1^ acetate buffer (pH 4.76) at [(CoTPRu_4_)_n_^8+^-GCE]/AuNPs^n−^ at different scan rates in the range of 20–200 mV s^−1^. *a*–*j*: 20; 40; 60; 80; 100; 120; 140; 160; 180; 200 mV s^−1^. Inset: plot of anodic and cathodic peak currents of CC versus square root of the scan rate.

A plot of *E*_pa_ versus pH from equation (3.1) (inside figure 5) provides a slope = −52. 5 mV pH^−1^, which is consistent with the theoretical value of −59 mV pH^−1^ [53], indicating that the redox processes of CC require the same number of protons and electrons. Figure 6 shows the result for CC detection, where the intensity of the redox peak currents increases linearly with the increase in the square root of the scan rate (*ν*^1/2^). Therefore, it indicates that the redox processes of CC on the surface of [(CoTPRu_4_)_n_^8+^-GCE]/AuNPs^n−^ are controlled by diffusion, according to equations (3.2) and (3.3):

3.2 $$I_{\mathrm{pa}}=(5.22\pm 0.09)\nu^{1/2}({\text{mV}}^{-2}\;{\text{s}}^{2})+(-6.69\pm 0.95)\;(R^{2}=0.9973),$$

3.3 $$I_{\mathrm{pc}}=(-3.25\pm 0.05)\nu^{1/2}({\text{mV}}^{-2}\;{\text{s}}^{2})+(2.67\pm 0.53)\;(R^{2}=0.9978).$$

### 3.4. Determination of catechol

**Figure 7.**
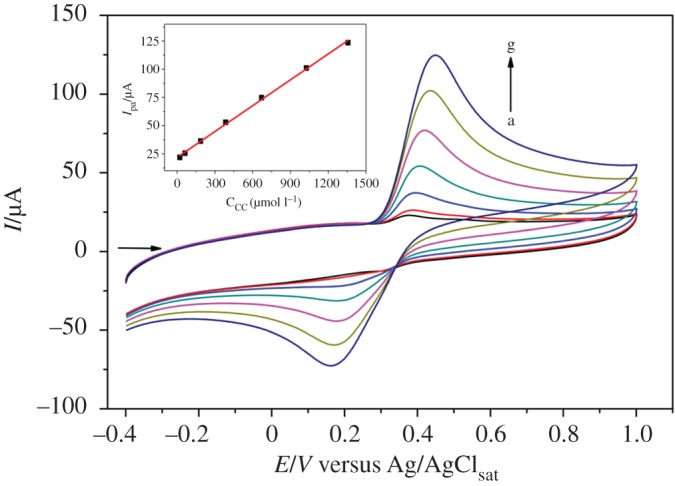
Cyclic voltammograms of CC as a function of concentration in the range of 21–1357 µmol l^–1^ in 0.1 mol l^–1^ acetate buffer solution (pH 4.76) at the [(CoTPRu_4_)_n_^8+^-GCE]/AuNPs^n−^ electrode. Inset: anodic peak currents versus CC concentration.

The determination of CC was carried out by CV using the [(CoTPRu_4_)_n_^8+^-GCE]/AuNPs^n−^. Figure 7 shows the cyclic voltammograms obtained in different concentrations of CC in 0.1 mol l^−1^ buffer acetate (pH 4.76), at a scan rate of 100 mV s^−1^.The results show a linear relationship between the *I*_pa_ and the CC (equation (3.4)) in a wide range of 21–1357 µmol l^−1^, with a detection limit of 1.4 µmol l^−1^ (S/N = 3) and the sensitivity of 108 µA µmol l^−1^ cm^−2^:

3.4 $$I_{\mathrm{pa}}(\mu \text{A})=7.64\;C_{\mathrm{CC}}(\mu \text{mol}\;{\text{l}}^{-1})+21.84\;(R^{2}=0.998).$$

The results suggest that the [(CoTPRu_4_)_n_^8+^-GCE]/AuNPs^n−^ electrode may be used for the determination of CC. The linear dynamic range (LDR), sensitivity (S) and detection limit (LD) of the proposed method were compared with other systems reported in the literature for the determination of CC (table 1). It can be observed that the proposed method shows good LD and an acceptable LDR, and the S of the [(CoTPRu_4_)_n_^8+^-GCE]/AuNPs^n−^ was greater than most of the reported electrochemical methods.

**Table 1.** Performance comparison of [(CoTPRu_4_)_n_^8+^-GCE]/AuNPs^n−^ electrode for the determination of CC with other modified electrodes reported in the literature.

electrode	technique	linear range (µM)	detection limit (µM)	sensitivity (µA µM cm^−1^)	ref.
[(CoTPRu_4_)_n_^8+^-GCE]/AuNP^n−^	CV	21–1357	1.4	108	this paper
VOTPRu-GCE	DPV	2–38	0.41	12.73	[46]
AuNPs-MPS/CPE	SWV	30–1000	1.1	—	[54]
GNPs/CNF/Au	DPV	5–350	0.36	—	[55]
Tyr-AuNPs-DHP/GCE	Amper.	2.5–95	0.17	115	[10]
Tyr-MWCNT-MNP/SPE	Amper.	10–80	7.61	4.8	[56]
Au-NP/HS(CH_2_)_6_SH-Au electrode	CV	2–8	—	—	[57]
GCE/{Nf-fc}-MME	CV	250–2500	10.8	1.1	[58]
MWCNT-GCE	DPV	20–1200	10	—	[59]
Au-GQDs/GCE	DPV	2–50	0.87	—	[60]
GO@PDA–AuNPs	DPV	0.3–77.5	0.015	4.66	[61]
CS/MWCNTs/PDA/AuNPs/GCE	DPV	0.1–10	0.047	—	[62]
GCE/Pt–MnO_2_	DVP	3–481	—	0.25	[17]
Ag-PGly/GCE	CV	0.6–100	0.1	—	[11]

CV = cyclic voltammetry; DPV = differential pulse voltammetry; SWV = square wave voltammetry, Amper. = amperometry technique.

### 3.5. Reproducibility and stability of the [(CoTPRu_4_)_n_^8+^-GCE]/AuNPs^n−^

The repeatability of [(CoTPRu_4_)_n_^8+^-GCE]/AuNPs^n−^ as a sensor to CC determination was evaluated by 20 successive cyclic voltammograms of a solution of CC (0.30 mmol l^−1^). The relative standard deviations of the *I*_pa_ was 1.4% after 20 measurements, suggesting that the [(CoTPRu_4_)_n_^8+^-GCE]/AuNPs^n−^ has a high level of reproducibility. Daily measurements were performed over a period of 15 days using the same electrode, conditioning the [(CoTPRu_4_)_n_^8+^-GCE]/AuNPs^n−^ before each analysis (see details in §2.3). As a result, the response of the [(CoTPRu_4_)_n_^8+^-GCE]/AuNPs^n−^ for CC detection was about 97% of its initial response obtained in the first day.

## 4. Conclusion

This work reports on the construction of an electronic device, using a bilayer based on AuNPs^n−^and tetraruthenated porphyrin, with the aim of applying it as an electrochemical sensor. Having the electrostatic interaction between the cationic polymeric film of porphyrin and anionic surface of AuNPs^n−^, the modified electrode based on self-assembly bilayers could be built, labelled as [(CoTPRu_4_)_n_^8+^-GCE]/AuNPs^n−^. Therefore, the number of bilayers and consequently the film thickness could be controlled. The performance of the [(CoTPRu_4_)_n_^8+^-GCE]/AuNPs^n−^ was evaluated in the determination of CC, where it exhibited well-defined voltammetric peaks with enhanced peak currents for the electrochemical process of CC, compared to the uncovered GCE or [(CoTPRu_4_)_n_^8+^-GCE]. Under the optimized conditions, a wide linear range for the detection of CC was obtained, from 21 to 1357 µmol l^−1^, and the detection limit was 1.4 µmol l^−1^ (S/N = 3). As far as we know, the [(CoTPRu_4_)_n_^8+^-GCE]/AuNPs^n−^ showed an exceptional sensitivity of 108 µA µmol l^−1^ cm^−2^ for CC, much higher than that of [(CoTPRu_4_)_n_^8+^-GCE] or other similar electrochemical sensors described in the literature. This exceptional sensitivity allows an accurate determination of CC concentration, that is ideal for an environmental samples analysis. In addition, it exhibits excellent stability over a period of 15 days, with a response of about 97%, owing to its initial response on the first day. The results suggest that the aggregation of AuNPs^n−^ for the polymeric film of porphyrin provides a significant increase in the electroactive area of the electrode.

## Data accessibility

All primary data collected and analysed for this study have been deposited with Figshare and can be accessed at https://doi.org/10.6084/m9.figshare.5514994.
